# Silent Cerebral Infarctions with Reduced, Mid-Range and Preserved Ejection Fraction in Patients with Heart Failure

**DOI:** 10.5935/abc.20180140

**Published:** 2018-09

**Authors:** Márcia Maria Carneiro Oliveira, Elieusa e Silva Sampaio, Jun Ramos Kawaoka, Maria Amélia Bulhões Hatem, Edmundo José Nassri Câmara, André Maurício Souza Fernandes, Jamary Oliveira-Filho, Roque Aras

**Affiliations:** 1 Escola de Enfermagem da Universidade Federal da Bahia, Salvador, BA - Brazil; 2 Hospital Cardio Pulmonar, Salvador, BA - Brazil; 3 Hospital Universitário Professor Edgard Santos (HUPES) - Universidade Federal da Bahia, Salvador, BA - Brazil; 4 Hospital Ana Nery - Universidade Federal da Bahia, Salvador, BA - Brazil; 5 Programa de Pós-graduação em Medicina e Saúde - Universidade Federal da Bahia, Salvador, BA - Brazil; 6 Ambulatório de Cardiomiopatias e Insuficiência Cardíaca - Universidade Federal da Bahia, Salvador, BA - Brazil

**Keywords:** Heart Failure, Cerebral Infarction, Stroke Volume, Stroke

## Abstract

Heart failure predisposes to an increased risk of silent cerebral infarction, and data related to left ventricular ejection fraction are still limited. Our objective was to describe the clinical and echocardiographic characteristics and factors associated with silent cerebral infarction in patients with heart failure, according to the left ventricular ejection fraction groups. A prospective cohort was performed at a referral hospital in Cardiology between December 2015 and July 2017. The left ventricular ejection fraction groups were: reduced (≤ 40%), mid-range (41-49%) and preserved (≥ 50%). All patients underwent cranial tomography, transthoracic and transesophageal echocardiography. Seventy-five patients were studied. Silent cerebral infarction was observed in 14.7% of the study population (45.5% lacunar and 54.5% territorial) and was more frequent in patients in the reduced left ventricular ejection fraction group (29%) compared with the mid-range one (15.4%, p = 0.005). There were no cases of silent cerebral infarction in the group of preserved left ventricular ejection fraction. In the univariate analysis, an association was identified between silent cerebral infarction and reduced (OR = 8.59; 95%CI: 1.71 - 43.27; p = 0.009) and preserved (OR = 0.05; 95%CI: 0.003-0.817, p = 0.003) left ventricular ejection fraction and diabetes mellitus (OR = 4.28, 95%CI: 1.14-16.15, p = 0.031). In patients with heart failure and without a clinical diagnosis of stroke, reduced and mid-range left ventricular ejection fractions contributed to the occurrence of territorial and lacunar silent cerebral infarction, respectively. The lower the left ventricular ejection fraction, the higher the prevalence of silent cerebral infarction.

## Introduction

Heart failure (HF) predisposes to an increased risk of cerebral abnormalities, including silent cerebral infarction, which is defined by the presence of infarctions (territorial or lacunar) in the brain parenchyma, verified through imaging methods, without a documented previous episode of stroke.^1,2^

The independent risk factors associated with silent stroke in HF are usually due to the impairment of left ventricular function, restrictive diastolic filling patterns in echocardiography, left atrial (LA) spontaneous echo contrast, and complex or calcified atherosclerotic aortic lesions.^3-5^

Ischemic stroke is a common complication of HF regardless of the Preserved (pLVEF) or reduced (rLVEF) Left Ventricular Ejection Fraction (LVEF).^5^ LVEF predicts the risk of cerebral infarctions, especially with rLVEF. It is believed that reduced blood flow may favor the formation of spontaneous echo contrast, intracavitary thrombi and consequent cardioembolic events.^6^ However, data explaining the stroke mechanism in HF in patients with LVEF are still limited,^7^ and data related to stroke and mid-range LVEF are scarce.

The objective of this study was to describe the clinical and echocardiographic characteristics and factors associated with silent cerebral infarction in patients with HF according to the LVEF groups.

## Methods

This is a prospective cohort performed at a referral hospital for the care of patients with HF in the city of Salvador, state of Bahia, Brazil, between December 2015 and July 2017. The diagnosis of HF was made according to the recommendations of the European Society of Cardiology (ESC),^8^ with patients who had signs and symptoms of HF, relevant structural heart disease (left ventricle (LV) body mass index ≥ 115 g in men and ≥ 95 g in women, or left atrial dilatation ≥ 40 mm) and or diastolic abnormality (E/A ratio < 0.75 or ≥ 1.5, or E-wave deceleration time < 140 ms). The LVEF groups were characterized as follows: rLVEF (≤ 40%), mid-range LVEF (mrLVEF; 41-49%) and pLVEF (≥ 50%).^9^ The diagnosis of Atrial Fibrillation (AF) was based on information available in medical records and the electrocardiogram.

### Assessment of cranial tomography

The cranial tomography was performed in all the patients to identify infarctions in the brain parenchyma (territorial or lacunar). The reports were analyzed by a neurologist, blinded to the patients' clinical data. These examinations were performed using a 1385 Toshiba Medical Systems Corporation device, (Shimo Ishigami, Otawara-Shi, Tochigi, Japan).

### Evaluation of transthoracic and transesophageal echocardiography

The examinations were performed by two experienced echocardiographists, as recommended by the American Society of Echocardiography (ASE).^10^ A commercially available device was used (Philips IE33, Philips Medical Systems, Andover, MA, USA), equipped with a 5 MHZ transducer with a multiplanar transesophageal probe. Subsequently, the images were recorded in a pen drive and reviewed by an echocardiographist.

The measures analyzed by the Transthoracic Echocardiogram (TTE) were: diastolic and systolic diameter of the LV, anteroposterior diameter of the LA, aortic root diameter, interventricular septum and posterior wall thickness. These analyses were obtained in the parasternal short axis and the parasternal long axis planes using the M-mode. The calculation of the LVEF was carried out using the LV modified biplane Simpson's method.

To perform the Transesophageal Echocardiography (TEE) images, the patient was placed in the left lateral decubitus position, and the left arm was extended over the head. The exams were performed under topical anesthesia with xylocaine spray at 10% and under intravenous sedation. The presence of spontaneous echo contrast and intracavitary thrombi were observed. The intracavitary thrombus was defined as an echodense intracardiac mass, and the spontaneous echo contrast was identified through its typical swirling movement, resembling smoke.^11^

### Statistical analysis

The collected data were processed using the Statistical Program for Social Sciences (SPSS), version 21.0. For data analysis, descriptive statistics were used (proportions and measures of central tendency), mean and standard deviation. The Kolmogorov-Smirnov was used in the normality test. The means and proportions were evaluated by Student's *t* test, according to the variable distribution. Pearson's chi-square test or Fisher's exact test was applied for association measures. Values were considered statistically significant when p ≤ 0.05 and the confidence interval ≥ 95%.

## Results

Seventy-five patients were studied. Comparisons of clinical and echocardiographic parameters are described in Table 1. The mean LVEF was 46 ± 16.5%. Spontaneous echo contrast and intracavitary thrombi were observed in the rLVEF group (19.3%), followed by mrLVEF (15.3%) and pLVEF (9.6%). Silent cerebral infarction was observed in 14.7% of the study population (45.5% lacunar and 54.5% territorial) and was detected more frequently in patients in the rLVEF group (29%), when compared to mrLVEF (15.4%, p = 0.005). There were no cases of silent cerebral infarction in the pLVEF group. In the univariate analysis, an association was identified between silent cerebral infarction and rLVEF (Odds Ratio - OR = 8.59, 95% of Confidence Interval - 95%CI: 1.71-43.27, p = 0.009) and pLVEF (OR = 0.05, 95%CI: 0.003-0.817, p = 0.003). There was no association with mrLVEF (OR = 1.07, 95%CI: 0.20-5.65, p = 0.936). The association of silent cerebral infarction with diabetes mellitus (OR 4.28; 95%CI: 1.14-16.15; p = 0.031) was also identified.

**Table 1:** Comparison of clinical and echocardiographic parameters between the groups of patients with heart failure with and without silent cerebral infarction

Parameters	Population n = 75	Silent cerebral infarctions		p value*
		Yes (n = 11)	No (n = 64)	
Age, years)	61.8 ± 10.6	62.5 ± 9.1	61.7 ± 10.9	0.817
Male gender	42 (56)	9 (81.8)	33 (51.6)	0.062
Arterial hypertension	60 (80)	8 (72.7)	52 (81.3)	0.514
Diabetes Mellitus	20 (26.7)	6 (54.5)	14 (21.9)	0.024
Ischemic heart disease	47 (62.7)	9 (81.8)	38 (59.4)	0.155
Permanent AF	13 (17.3)	3 (27.3)	10 (15.6)	0.346
**NYHA class =**				
I	20 (26.7)	2 (18.2)	18 (28.1)	0.491
II	41 (54.7)	7 (63.6)	34 (53.1)	0.518
III	14 (18.7)	2 (18.2)	12 (18.8)	0.964
**HF etiology**				
Idiopathic	33 (44)	3 (27.3)	30 (46.9)	0.226
Chagasic	27 (36)	5 (45.5)	22 (34.4)	0.479
Ischemic	10 (13.3)	2 (18.2)	8 (12.5)	0.609
Hypertensive	3 (4)	1 (9.1)	2 (3.1)	0.351
Valvar	1 (1.3)	-	1(6.9)	0.676
Rheumatic	1 (1.3)	-	1 (1.6)	0.676
**LVEF subgroups**				
Reduced (≤ 40%)	31 (41.3)	9 (81.8)	22 (34.4)	0.003
Mid-range (41-49%)	13 (17.3)	2 (18.2)	11 (17.2)	0.936
Preserved (≥ 50%)	31 (41.3)	0 (0)	31 (48.4)	
**Echocardiographic data**				
LA diameter, mm	43.9 ± 8.9	46.2 ± 10.6	42.9 ± 8.5	0.264
LV dilatation	31 (41.3)	8 (72.7)	23 (35.9)	0.022
**Intracavitary thrombi/ spontaneous echo contrast**				
Intracavitary thrombi / spontaneous echogenic contrast in LA	9 (12.1)	1 (9.1)	8 (12.5)	0.552
Intracavitary thrombi / spontaneous echo contrast in LAA	2 (2.6)	1 (9.1)	1 (1.6)	0.351
**Medications**				
Aspirin	41 (54.7)	6 (54.5)	35 (54.7)	0.993
Warfarin	13 (17.3)	1 (9.1)	12 (18.8)	0.434
NOAC	6 (8)	2 (18.2)	4 (6.3)	0.178

Results expressed as mean ± standard deviation or n (%).

* Student’s t test for categorical variables and Pearson’s chi-square for continuous variables. AF: arterial fibrillation; NYHA: New York Heart Association; HF: heart failure; LVEF: left ventricular ejection fraction; LA: left atrium; LV: left ventricle; LAA: left atrial appendage; NOAC: new oral anticoagulants.

## Discussion

In our study, patients with rLVEF had silent cerebral infarction in the territory region, and those with mrLVEF had silent cerebral infarction of the lacunar type. There was no silent cerebral infarction in patients with pLVEF. It was demonstrated that the lower the LVEF, the higher the prevalence of silent cerebral infarction. A previous study showed that reduced LVEF values are associated with patients with silent stroke (p = 0.030).^12^

The prevalence of silent cerebral infarction in this study was considered small when compared to other studies of HF patients. In a study of 117 patients with HF evaluated for heart transplant, the prevalence of ischemic stroke was 34%.^13^ In the study by Kozdag et al.,^12^ with 72 patients with ischemic dilated cardiomyopathy, the prevalence of silent cerebral infarction was 39%. However, it is worth mentioning that the high prevalence of silent infarctions in these studies was probably the result of increased HF severity in the studied populations.

Another important finding was the association between diabetes mellitus and silent cerebral infarction. Chen et al.^14^ found that abnormalities in early LV diastolic filling were commonly observed in diabetic patients, and the proposed mechanism includes, among other factors, microvascular disease, which may justify the data found in our study.

In the case of patients with mrLVEF, silent cerebral infarctions were reported to be of the lacunar type, usually associated with cerebral small vessel disease, but eventually of embolic etiology.^15^ A recent study clearly demonstrated that the clinical characteristics of mrLVEF are intermediate between pLVEF and rLVEF, or close to pLVEF or rLVEF, and suggest that mrLVEF is a transitional stage from pLVEF to rLVEF, or from rLVEF to pLVEF, rather than a distinct HF class.^16^ However, data are still limited regarding these patients.

Patients in the LVEFp group did not show silent cerebral infarction, differently from a study on LVEF groups, in which the rates of stroke or transient ischemic attack were slightly higher in patients with pLVEF vs. patients with rLVEF and mrLVEF. It is worth mentioning that AF was more common in these patients with pLVEF, although the AF was associated with an increased risk of stroke or transient ischemic attack, regardless of LVEF status.^17^

## Conclusion

In patients with heart failure and without a clinical diagnosis of stroke, the reduced and mid-range left ventricular ejection fractions contributed to the occurrence of territorial and lacunar silent cerebral infarction, respectively. In cases of preserved left ventricular ejection fraction, there was no prevalence of silent cerebral infarction; reduced left ventricular ejection fraction and diabetes mellitus were associated with embolic cerebral infarction, and the lower the left ventricular ejection fraction, the higher the prevalence of silent cerebral infarction. Further studies are required to elucidate the mechanisms of silent cerebral infarction in the left ventricular ejection fraction groups.

### Limitations

The study was carried out in a single center, with a small sample and there were no analyses of intra- and interobserver variability between the echocardiographists.
